# Participation in Play and Leisure Activities of Young Children with Autism Spectrum Disorder and Typically Developing Children in Taiwan: A Preliminary Study

**DOI:** 10.3390/ijerph18115787

**Published:** 2021-05-27

**Authors:** Chi-Ching Huang, Lin-Ju Kang

**Affiliations:** 1 Graduate Institute of Early Intervention, College of Medicine, Chang Gung University, Kwei-Shan, Tao-Yuan 33302, Taiwan; cchuang79@gmail.com; 2 Department of Physical Medicine and Rehabilitation, Chang Gung Memorial Hospital, Linkou 333423, Taiwan

**Keywords:** participation, autism spectrum disorder, children, play, recreation, leisure

## Abstract

Participation in enjoyable activities is essential for the health and development of young children with and without disabilities. For preschool children with autism spectrum disorder (ASD), there is limited knowledge regarding their participation in play, learning, recreation, and social activities. This was a preliminary study that compares the participation between children 2–6 years of age with ASD (*n* = 25) and age- and sex-matched typically developing (TD) (*n* = 25) children. The Chinese version of the Assessment of Preschool Children’s Participation (APCP-C) measures participation in play, skill development, active physical recreation, and social activities. Parents of the children in this study completed the APCP-C by structured interview. The results showed that children with ASD had lower participation diversity and intensity than TD children in play activities. A lower percentage of children participating in individual activity was found for children with ASD than TD children in most APCP-C activities. Professionals that serve young children with special needs are encouraged to partner with parents to provide playful and socially enhancing activities for preschool children with ASD.

## 1. Introduction

Participation in enjoyable and meaningful activities is essential for the health and development of preschool children both with and without disabilities [1]. The International Classification of Functioning, Disability and Health defines participation as the nature and extent of involvement in life situations [2]. A synthesis of research indicated that participation involves attendance and involvement as two essential components [3,4]. Attendance refers to being present in activities, and involvement refers to the experiences while attending activities [4]. In this study, we focus on exploring the attendance aspects of participation, as it is a starting point to provide opportunities for learning and development [5], building social friendships [6], and developing a sense of self-identity [7]. Therefore, participation is essential for early childhood development, particularly for children with disabilities and including them in their communities.

Autism spectrum disorder (ASD) is a complex, lifelong developmental condition that can cause various social, communication, cognitive, and behavioral challenges [8,9]. Previous researchers have identified the core impairments of ASD as being in communication impairments, social deficits, sensory differences, and restrictive, repetitive, and stereotyped behaviors, which can affect children’s engagement in play and recreational activities [10,11]. Atypical ways of thinking, moving, interacting, and sensory processing, as well as the repetitive and restricted behaviors including sensory sensitivities, insisting on sameness, and dislike of change, might limit the interests and activity selection of children with ASD [12]. In a national survey study in Taiwan, children with ASD showed less independence and less frequent participation than did children with other types of disabilities (intellectual, physical, language) [13].

There is little available knowledge regarding young children with ASD, particularly their participation in daily activities specifically related to play, recreation, and socialization. There have been even fewer comparisons between children with ASD and their typically developing (TD) peers. LaVesser and Berg compared participation patterns in preschool children with and without ASD and reported that children with ASD participated in significantly fewer activities than did TD children in activities related to self-care, community mobility, leisure, social interaction, chores, and education. Parents of children with ASD reported that the primary reason for children’s nonparticipation was behavioral difficulties [14]. In a small sample study, Rodger and Umaibalan reported that families of children with ASD engaged less in family rituals such as special celebrations, annual celebrations, weekends, and vacations than did the families of TD children. Limited participation in these rituals reduced families’ opportunities to enjoy free-time activities, interact with family members, and expand social networks [15]. No researchers have studied in depth the participation of preschool children in a broader set of play, skilled, physical, and social activities.

Despite the importance of participation in young children, studies of children with ASD have focused on children over 6 years of age [16]. Many researchers have assessed the diversity and intensity of leisure and recreational participation as part of the Children’s Assessment of Participation and Enjoyment (CAPE) [17], and children with ASD have been reported to participate in fewer recreational and leisure activities than TD children [11,18,19], especially social [18] and physical [11,18] activities. Children with ASD also showed less participation than TD children, especially in social and unstructured leisure activities [18].

Research has reported more intense participation in quiet recreational activities among children with ASD [11,18]. These children tended to spend time on quiet recreation such as watching television and playing with toys or video games [20], and they tended to do activities at home alone or with family members [18]. In all these studies, although TD children and children with ASD participated in a variety of activities, school-age children with ASD showed different diversity and intensity of participation from that of TD children. However, it is unclear whether participation differs between preschool children with and without ASD. More importantly, most previous studies have been conducted in Western countries. Research in the Chinese cultural context, such as Taiwan, is quite limited.

The Assessment of Preschool Children’s Participation (APCP), modeled after the CAPE, is a parent-completed measure of the diversity and intensity of participation of children 2–6 years of age [21]. The APCP categorizes activities as play, skill development, active physical recreation, or social activities that young children usually participate in with their families or in their communities. The APCP has successfully revealed patterns of participation in children with and without childhood disabilities in various cultures [22,23,24], and authors of a scoping review suggested using it to assess the participation of preschool children with ASD [25].

To gain a better understanding of the participation of preschool children with ASD, it is important to identify their patterns of participation in daily activities compared with the patterns observed among preschool TD children. Age [21,22,26] and sex [21,22,27,28] are two potentially important personal factors that might affect participation, this study is designed to compare children with ASD with their age- and sex-matched peers. The purpose of this preliminary study was to identify the similarities and differences in participation between children with ASD and TD children in Taiwan. The two specific research questions were as follows:

(1) Do the diversity and intensity of participation in play, skill development, active physical recreation, and social activities differ between children with ASD and age- and sex-matched TD children?
(2) Does the percentage of children participating in individual activity differ between children with ASD and TD children?

## 2. Materials and Methods

### 2.1. Participants

Both the children with ASD and the TD children were recruited from the northern area of Taiwan using convenience sampling. Our eligibility criterion for children with ASD was a primary Diagnostic and Statistical Manual, Fifth Edition diagnosis of ASD (American Psychiatric Association, 2013). TD children were those who did not have any diagnosis or conditions related to developmental delays or disabilities. Either a pediatrician or a pediatric psychiatrist made the diagnosis and assessed the severity of the children’s impairments (i.e., mild, moderate, severe, and profound) based on the grading rules developed in the ICF-based Disability Evaluation System (DES) [29,30] and through joint assessment by a multidisciplinary team. The children’s diagnoses and severity levels were obtained from their medical records. The children’s diagnoses and severity levels were confirmed with their medical records. The children’s age was between 2 and 6 years old when they began participating in the study, and the two groups were matched by age and sex. Children with ASD were recruited by parental or professional referrals from preschool special education services in northern Taiwan, and the TD children were recruited from regular preschools. Ethical approval was provided by the institutional review board of a medical center in northern Taiwan. Informed consent was obtained from all parents of the children included in the study.

The study comprised a total of 50 children: 25 children diagnosed with ASD and 25 age- and sex-matched TD children (mean age = 4.9; SD = 0.9; range = 2–6 years old). Among the 25 children with ASD, 12 had other diagnoses (attention-deficit hyperactivity disorder, language delay, and developmental delay) as well. The children with ASD showed symptoms of varying levels of severity: mild (*n* = 9), moderate (*n* = 9), severe (*n* = 5), and profound (*n* = 2). Table 1 presents the characteristics of the participating children and their parents. We used the functional skills scales on the Chinese version of the Pediatric Evaluation of Disability Inventory (PEDI-C) to measure the children’s capabilities in daily situations [31]. The scores were scaled to range from 0 to100, and higher scores indicate higher functionality. According to the scores, children with ASD showed significantly lower self-care and social functionality, such as with communication, problem-solving, and social interaction, compared with TD children (*p <* 0.001). The majority of children in both groups attended preschools, although eight children with ASD attended an early intervention institute. The parents’ education levels in both groups were not statistically significantly different, but the fathers’ education attainment was lower for children with ASD than for TD children (20% vs. 48% who had graduate degrees).

### 2.2. Instruments

#### Chinese Version of the Assessment of Preschool Children’s Participation (APCP-C)

The APCP-C was used as the main measure of children’s participation patterns. It contains 45 items that assess preschool children’s participation in four types of activities: play (9 items, e.g., “playing with toys”), skill development (15 items, e.g., “learning to dance”), active physical recreations (10 items, e.g., “doing water activities”), and social activities (11 items, e.g., “going to a party”). The list of all items is available in Table 2. For the diversity score, parents first answer “yes” or “no” regarding whether the child has participated in the activity over the past four months, and for yes responses, intensity was measured by asking parents to score how often the child had participated in the activity for the past four months using a seven-point scale (1 = once over the past four months to 7 = once a day or more). The APCP-C was translated into traditional Chinese and culturally adapted from the original English version [21]; researchers have reported on the translation process, pilot testing, and psychometric properties [23]. Researchers calculated excellent internal consistency (Cronbach’s α = 0.85 and 0.86) and test–retest reliability (ICCs = 0.79) for both diversity and intensity for all 45 items together; reliabilities varied from excellent to poor for activity scores, and initial evidence for cross-cultural validity and construct validity was also established [23]. We analyzed both diversity and intensity for the four activity scores in this study.

### 2.3. Procedure

After parents agreed to participate in the study, a trained interviewer contacted them to schedule an in-person interview. Before collecting any data, the interviewer received a training course including the concepts, evidence, administration, and scoring of the PEDI-C and APCP-C. Parents were interviewed either in their homes or at their preferred locations, first completing a demographic questionnaire. We designed the demographic questionnaire for this study to document basic information on the children and their families, including children’s age, sex, and primary diagnosis and parents’ age, education level, and employment status. Parents then completed the APCP-C by structured interview during which the trained interviewer asked each parent each item on the scale. The benefit of an in-person structured interview was that the interviewer was able to clarify any of the parents’ concerns about wording or examples.

### 2.4. Data Analysis

We analyzed the data for the study with SPSS 26.0 (IBM Corporation, Armonk, NY, USA). We calculated the APCP-C diversity and intensity scores for each of the four activity types. We calculated a diversity score by adding the children’s scores for each activity they have done over the past 4 months. That is, a higher diversity score indicates a wider variety of activities the child has engaged in. Because there were different numbers of activities in the different types, to enable comparing activity types, we converted the diversity scores to percentages (%) by dividing the sum score by the number of items in each activity type.

We calculated an intensity score by dividing the sum of frequencies for all items by the number of possible items in each activity type; if the child did not do the activity in the past four months, the score was 0. The total scores ranged from 0 to 7. We used the chi-square test to examine the group differences in characteristics of the participating children and their families: child’s education status and parents’ education level, employment status, and household income. We also used independent t tests to examine the group differences in the PEDI-C scores as well as to compare the participation between the ASD and TD groups.

We calculated the effect size (Cohen’s *d*) to examine the magnitude of differences. According to Cohen (1988), *d* = 0.2 is a small effect size, *d* = 0.5 is medium, and *d* = 0.8 is large. Due to the number of analyses we conducted, we set significance at *p* = 0.01 to reduce the possibility of a type I error [32]. The percentage of children participating in individual activity was calculated from the numbers of children who were reported to perform the activity divided by the total number of children in each group. This approach was useful for visualizing distinct participation patterns at the item level between children with and without ASD [11]. The activities were ranked by the percentage of the ASD group in descending order.

## 3. Results

### 3.1. Participation Diversity and Intensity of Children with ASD Versus TD

Table 3 presents the results for participation diversity and intensity of the APCP-C. For the four types of activities, there was only a significant difference in play activities between the two groups (*p* < 0.001) with a large effect size (*d* = 1.3); no significant differences were found in the skill development, active physical recreation, and social activities activity types. In addition, the participation diversity calculated as percentages showed some variations among different activity types. The ASD group had participated most diversely in skill development activities followed by play, physical, and social activities. The TD group had participated most diversely in play activities followed by skill development, physical, and social activities. Activity type with higher percentages means children experienced a greater variety of items in this activity type.

For the four types of activities, only play activity showed a significant difference between the two groups (*p* < 0.001) with a large effect size (*d* = 1.2). No significant differences were found for the other three types of activities.

### 3.2. Percentage of Children Participating in Individual Activity

To gain a better understanding of the differences in participation between children with and without ASD, we performed item-by-item analyses. Table 2 presents the APCP-C items and percentages of children participating in each activity for each group; items were organized by activity type and ranked in descending order based on the percentage of children in the ASD group. In general, higher percentages of children in the TD group had experienced these individual activities of the APCP-C than children in the ASD group. The top activities that most children participated in were different between the two groups. There was a total of 11 activities that 100% of the children in the TD group had participated in the last 4 months, with five activities in each type of play and skill development and one in active physical recreation. In contrast, we found no activities that all children in the ASD group had participated, although there were six activities that 96% of the children in the ASD group had participated in, with three skill development activities and one each in play, active physical recreation, and social activities.

Despite an overall pattern of higher percentages of participating children in the TD group, there were a total of seven activities that children in the ASD group participated at least 10% more than the TD group, four in skill development, two in active physical recreation, one in social activities, and none in play.

## 4. Discussion

### 4.1. Similarities and Differences in Participation Diversity and Intensity

This study revealed that in the Taiwan context, both children with ASD and TD explored a variety of play, skill development, active physical, and social activities. However, children with ASD had participated in less diverse and less intensive play activities than had TD children. Our findings are supported by the evidence of differences in play behaviors between children with ASD and their peers [28,33]. For example, Harrop et al. (2017) studied play complexity and toy engagement in a laboratory setting in which play complexity was categorized into simple object play, combination play, presymbolic play, and symbolic play [34]. Their results showed that children with ASD spent less time in presymbolic play and spent more time not engaged in play behaviors or with toys than did TD children [28]. This corresponds with our findings that parents reported less diverse and intense daily play activities among children with ASD than among TD children. Engagement in play activities and the social contexts of these activities enhance the learning of young children and results in benefits on physical, cognitive, and socioemotional development [35,36]. However, preschool children with ASD are less likely to engage in free play with peers, while TD children are usually engaged jointly with peers [37]. It is also possible that children with ASD need to spend time in taking therapy sessions, thus limit their time and opportunities in free play.

We found no differences in participation diversity and intensity in skill development, physical, and social activities in the current study; children with ASD showed similar participation in skillful, physical, and social activities with their peers. The nature of activities may explain this result. Unlike play activities that are usually initiated by children, skill development, physical, and social activities are more likely to be influenced by family choices and opportunities available within people’s homes and communities. Activities such as taking music lessons, playing physical games, or going on full-day outings often require arrangements by adults. It is recognized that children at preschool age usually participate in activities within their families but that as children grow, differences in participation may be shown in multiple types of activities (e.g., physical, recreational, and social activities) as suggested by previous studies involving school-age children with ASD [11,18,19]. Children with ASD are likely to face increased challenges in organized social or physical activities when they enter school age.

### 4.2. Similarities and Differences in Participation in Individual Activity

In general, the ranking order of the percentages of participation was comparable between children with ASD and TD, meaning that the most and least commonly performed activities were quite similar for children with ASD and TD children. However, we identified less participation among children with ASD in most of the individual play, skill development, active physical recreation, and socialization activities. In the results, fewer children with ASD had participated in these activities in the past four months than their TD peers, as found in previous studies [11,38]. Limited interests and exploration especially in spontaneous and unstructured settings might explain the participation restriction of children with ASD [39].

Despite a general pattern of lower participation in children with ASD, there were seven activities in which children with ASD had a higher percentage of participation by at least 10% than TD children, namely, doing gymnastics, painting, taking music lessons, taking swimming lessons, going for walks, doing water activities, and playing computer games. Parents of children with ASD but not of TD children often reported gymnastics participation and attending physical and/or occupational therapy sessions for children. Painting, music lessons, swimming lessons, or water activities were usually reported as formal learning opportunities arranged by parents to facilitate skill development or expand interests for children with ASD. Playing computer games was more commonly performed by children with ASD than TD children, consistent with the findings for school-age children [11]. Though we did not further investigate how children use computer products, parents should be aware of their children’s screen time and the impact of computer products on their behaviors.

### 4.3. Limitations and Future Directions

This study involved a convenience sample with a small sample size, thus limiting the generalizability of this study. We collected our data through parent reports and interviews and verified the diagnosis of ASD through medical records provided by parents but not diagnostic assessments. No observation data while children attending the activities were collected in this study. In addition, we did not use standardized tests for intelligence and language. We measured children’s self-care and social functions with the PEDI-C. As skills to managing daily livings and abilities in social interaction may impact children’s activity choices and involvement in play and leisure activities [20], further analysis could consider the PEDI-C scores as covariates of participation diversity or intensity. Our preliminary study findings suggest the need for a larger comparative study. The effect sizes reported in this study will help estimate the sample size needed to detect differences in participation between young children with ASD and TD in future studies. Additionally, the APCP-C captures the frequency of attendance; children’s interests, activity preferences, or level of involvement in an activity are not included. As a result, the use of diagnostic and developmental assessments applicable to ASD, and a measure including both participation attendance and involvement [40], is suggested to provide a complete profile of participation of young children with ASD.

### 4.4. Implications for Practice

Preschool age is a critical time to provide children with opportunities to explore and enhance participation in various kinds of activities in their homes and communities. The results of this study draw attention to the restriction in play participation experienced by young children with ASD. Free play activities such as playing with toys and pretend or imagery play are usually initiated by children and depend on the child’s interest and initiative. Evaluation of preschool children with ASD is important for including the variability and frequency of participation, as is considering the complexity and social involvement in play [41]. The APCP-C diversity and intensity scores capture the breadth of play and leisure activities in which a child attends, and the frequency of attendance. Children need to have the opportunity to attend an activity before they can further develop personal experiences from involvement in the activity [4]. Attendance (e.g., diversity and frequency of participation) and involvement (e.g., motivation, play complexity, and social engagement) in play activities should be a focus when planning interventions for preschool children with ASD.

Separately, participation is closely related to children’s surrounding environments and resources. In this study, fathers’ educational attainment was lower among children with ASD, which may be related to the accessibility and availability of family resources. Family and peer support are important facilitators of participation by children with ASD [42]. Increasing community supportiveness of children with ASD may also facilitate participation of children with ASD and decrease caregiver’s feelings isolation [43]. It is critical to ensure that programs or services for play and leisure are available, accessible, accommodative, and acceptable to better support inclusion of children with ASD and their families. Professionals who work with young children with special needs are encouraged to recognize the importance of play participation, partner with parents and community members, and provide services or resources to foster playful and socially enhancing activities.

## 5. Conclusions

This study revealed patterns of participation of young children with ASD in comparison with their age- and sex-matched TD peers. Both groups of children participated in a variety of play, skilled, physical, and social activities. However, children with ASD had lower participation diversity and intensity than their TD peers, especially in the area of play. Looking at individual activity, the most and least commonly participated activities were similar for children with ASD and TD. However, fewer children with ASD participated in most of the activities. Further investigation of how children involve themselves in or enjoy these activities is necessary to provide a picture of the actual daily life experiences of these children.

## Figures and Tables

**Table 1:** The Characteristics of Participants.

Variables	ASD (*n* = 25)	TD (*n* = 25)	*p* ^a^
Child’s age, *n* (%)			
2–4 years	13 (52)	13 (52)	1.000
5–6 years	12 (48)	12 (48)	
Child’s sex, *n* (%)			
Boy	21 (84)	21 (84)	1.000
Girl	4 (16)	4 (16)	
PEDI-C scaled score, Mean (SD)			
Self–Care	55.9 (10.7)	78.4 (16.3)	<0.001
Mobility	88.6 (9.3)	93.4 (8.3)	0.093
Social Function	54.0 (18.3)	87.8 (11.4)	<0.001
Child’s schooling, *n* (%)			
Preschool	16 (64)	24 (96)	0.008
EI institute	8 (32)	0 (0)	
Did not attend school	1 (4)	1 (4)	
Father’s education level, *n* (%)			
Junior high school and under	1 (4)	0 (0)	0.191
High school graduate	4 (16)	4 (16)	
College/University graduate	13 (52)	9 (36)	
Graduate degree	5 (20)	12 (48)	
Unanswered	2 (8)	0	
Mother’s education level, *n* (%)			
Junior high school and under	2 (8)	0	0.776
High school graduate	4 (16)	4 (16)	
College/University graduate	17 (68)	16 (64)	
Graduate degree	5 (20)	5 (20)	
Unanswered	2 (8)	0	

Note. ASD, Autism spectrum disorder; TD, typical development; EI, early intervention; PEDI-C, Chinese version of Pediatric Evaluation of Disability Inventory. ^a^ *p* values were analyzed by Independent t tests for PEDI-C scores, and from Chi-square tests for other characteristics.

**Table 2:** Comparison of Percentage of Children Who Experienced Each Activity between Children with ASD and TD.

Activities	ASD (%)	TD (%)
Play activities		
Playing with toys	96	100
Exploring	92	100
Watching TV or a video	88	100
Playing a musical instrument	88	92
Creating a craft project	80	100
Doing pretend or imaginary play	72	100
Building forts or tents	52	84
Collecting things	32	96
Playing with pets	28	28
Skill development		
Doing gymnastics	96	64
Drawing and coloring	96	100
Doing a puzzle	96	92
Building things	92	100
Listening to stories	84	100
Cutting and pasting	84	96
Painting	80	68
Helping around the house	80	100
Reading or looking at books	76	100
Taking music lessons	76	56
Learning to dance	72	84
Participating in religious activities	56	76
Picking out books and movies	48	84
Taking swimming lessons	32	4
Participating in community organization	20	36
Active physical recreation		
Playing on playground equipment	96	100
Dancing	88	84
Interacting with nature	88	96
Playing physical games	88	92
Riding a bicycle, tricycle, or scooter	88	92
Going for walks	80	68
Doing water activities	64	40
Gardening	16	60
Doing team sports	12	28
Doing snow activities	0	8
Social activities		
Going on a full or half day outing	96	96
Listening to music	88	88
Playing computer games	80	60
Going to a party	76	92
Playing board or card games	56	60
Going to a live event	48	56
Backing and cooking	44	76
Playing dress up	44	68
Attending a play group	36	40
Having someone over to play	20	20
Going to the movies	20	32

Notes: ASD, Autism spectrum disorder; TD, typical development. Bolded activities with percentages are those that at least 10% higher percentage of participation in group ASD than TD group.

**Table 3:** Comparisons of APCP-C Mean Scores between Children with ASD and TD Children.

Domains	ASD	TD	*T*	Cohen’s
(Number of Items)	M (SD), %	M (SD), %		*d*
Participation diversity				
Play (9 items)	6.3 (1.7), 70	8.0 (0.8), 89	4.6 *	1.3
Skill-development (15 items)	10.9 (2.1), 73	11.5 (2.0), 77	1.0	0.3
Physical (10 items)	6.2 (1.3), 62	6.7 (1.3), 67	1.3	0.4
Social (11 items)	6.1 (1.9), 56	6.8 (2.2), 62	1.1	0.3
Participation intensity				
Play (9 items)	3.8 (1.1)	5.0 (0.9)	4.3 *	1.2
Skill-development (15 items)	3.7 (0.8)	4.0 (1.1)	1.3	0.3
Physical (10 items)	3.1 (0.7)	3.3 (0.9)	1.0	0.2
Social (11 items)	2.3 (0.8)	2.2 (0.8)	−0.5	0.1

Note. ASD, Autism spectrum disorder; TD, typical development. * Significant difference at *p* < 0.01 two tailed Cohen’s *d*: 0.2 = small effect, 0.5 = medium effect, 0.8 = large effect % was calculated by dividing the dividing the mean scores with the number of items in each type of activity.

## Data Availability

The data presented in this study are available on request from the corresponding author. The data are not publicly available due to ethical issues.
